# The Ability of Different Imputation Methods to Preserve the Significant Genes and Pathways in Cancer

**DOI:** 10.1016/j.gpb.2017.08.003

**Published:** 2017-12-13

**Authors:** Rosa Aghdam, Taban Baghfalaki, Pegah Khosravi, Elnaz Saberi Ansari

**Affiliations:** 1 School of Biological Science, Institute for Research in Fundamental Sciences (IPM), Tehran 19395-5746, Iran; 2 Department of Statistics, Faculty of Mathematical Sciences, Tarbiat Modares University, Tehran 14115-111, Iran; 3 Department of Physiology and Biophysics, Institute for Computational Biomedicine and Institute for Precision Medicine, Weill Cornell Medical College, New York, NY 10021, USA; 4 Institut Cochin, Inserm U1016, CNRS UMR 8104, Universit Paris Descartes UMR-S1016, F-75014 Paris, France

**Keywords:** Gene expression, Missing data, Imputation method, Significant genes, Pathway enrichment

## Abstract

Deciphering important genes and pathways from incomplete **gene expression** data could facilitate a better understanding of cancer. Different **imputation methods** can be applied to estimate the missing values. In our study, we evaluated various imputation methods for their performance in preserving **significant genes** and pathways. In the first step, 5% genes are considered in random for two types of ignorable and non-ignorable missingness mechanisms with various missing rates. Next, 10 well-known imputation methods were applied to the complete datasets. The significance analysis of microarrays (SAM) method was applied to detect the significant genes in rectal and lung cancers to showcase the utility of imputation approaches in preserving significant genes. To determine the impact of different imputation methods on the identification of important genes, the chi-squared test was used to compare the proportions of overlaps between significant genes detected from original data and those detected from the imputed datasets. Additionally, the significant genes are tested for their enrichment in important pathways, using the ConsensusPathDB. Our results showed that almost all the significant genes and pathways of the original dataset can be detected in all imputed datasets, indicating that there is no significant difference in the performance of various imputation methods tested. The source code and selected datasets are available on http://profiles.bs.ipm.ir/softwares/imputation_methods/.

## Introduction

Cancer has manifested as one of the major health problems in many countries worldwide. It is also expected to be the main cause of death in the next few years [1]. Cancer has been characterized as a heterogeneous disease, comprising various subtypes. Early diagnosis of the cancer type and stage has become essential to assist with the subsequent treatment of cancer patients [2]. With the technical advances in sequencing, it is now possible to measure the expression of all genes in a sample and stratify cancer patients into high-risk and low-risk cohorts by analyzing gene expression data using bioinformatics approaches [3].

Recognizing the genes involved in cancer is an intimidating challenge due to its importance in the molecular characterization of widely defined biological classes, which has a potential role in cancer diagnosis and treatment. The growing application of bioinformatics approaches in cancer encourages researchers to develop newer techniques involving the whole genome-based microarray. The gene expression datasets, as well as many other real-world datasets, often contain missing values, thereby affecting the inference of significant genes and the associated pathways or networks. There are many reasons for the occurrence of missing values in microarray gene expression data, *e.g.*, hybridization failures, low resolution, artifacts on the microarray, image noise, corruption, and spotting problems [4], [5], [6], [7].

Mechanically, missing values can be classified as missing completely at random (MCAR), missing at random (MAR), and not missing at random (NMAR) [8]. MCAR and MAR are considered ignorable, whereas NMAR is considered non-ignorable or informative missingness. Identifying the appropriate missing mechanism and missingness rate is important for imputation algorithms [9].

For microarray gene expression datasets, there are global, local, and hybrid imputation approaches, categorized according to the information used in each case [5]. The global missing imputation methods exploit the global information of the whole dataset, whereas the local missing imputation methods use the local similarity structure of a dataset. Hybrid methods combine the two to impute missing values.

Previous studies have shown that a missingness of ≤1% in expression data is negligible and a missingness of 1%–5% is manageable. To achieve good results in imputation for an incomplete dataset with 5%–15% missingness, it is important to use appropriate approaches. When datasets have >15% missing data, choosing imputation methods may strongly influence the results [5].

Therefore, we set out to investigate the impact of missingness factors on the imputation algorithms and evaluated the performance of 10 popular imputation methods by applying five well-known methods to acquire the significant genes from the original and imputed datasets for lung and rectal cancers. Our results indicate that similar important genes are detected in all imputed datasets, suggesting no significant difference in the performance of the imputation methods tested in terms of preserving the essential genes and pathways.

## Methods

### Data sources

Whole genome-based microarray data were downloaded from the Gene Expression Omnibus (GEO) database [10] with accession number GSE10072 [11] and GSE15781 [12] for lung and rectal cancer, respectively. The lung cancer dataset contains 107 samples from 58 patients with lung cancer and 49 healthy individuals, whereas the rectal cancer dataset contains 42 samples from 22 patients with rectal cancer and 20 healthy individuals. The linear model for microarray analysis (Limma) package in R [13] was used for preprocessing and analysis of the microarray data. Quantile normalization [14] is then performed to achieve the same sample distribution at each state.

### Data processing for generation of missing values

The gene expression datasets often contain a small proportion of genes with missing values [5]. To generate missing values in a dataset, 5% of all genes from the original datasets were selected randomly in the first step of our study. Then, ignorable and non-ignorable types of missingness were considered at a missingness rate of 10%, 20%, and 30%, respectively. To generate ignorable missing values, the samples were randomly selected based on the three rates of missingness, and then were removed. Furthermore, to generate non-ignorable missing values, the upper or lower tails (10%, 20%, and 30%) of the data were selected, and their values were removed to ensure that the missingness depends on the actual gene expression.

### Imputation methods

Ten imputation methods are considered in this study. Among them, the singular value decomposition (SVD), the Bayesian principal component analysis (BPCA), fast imputation (Fast-Imp), column-mean, column-median, gene-mean, and gene-median are global methods, whereas local least squares (LLS) and K-nearest neighbor (KNN) are local methods. Multiple imputation by chained equations and classification and regression trees (MICE-CART) is a hybrid method.

The SVD imputes missing values using the singular value decomposition and regression models [15]. The k genes similar to a target gene, which contains missing values, are detected by KNN method using a similarity metric calculated with the non-missing data. Then, the weighted average of these neighbors is calculated to impute the missing values in target gene [15]. The MICE-CART imputation method encloses MICE and CART approaches [16]. Principle component regression, an expectation–maximization (EM) algorithm, and the Bayesian estimation approach are applied in the BPCA imputation method [17]. In order to impute the missing values, a multiple regression model is applied in LLS method [18]. The EM algorithm under the multivariate normal distributional assumption is used in a Fast-Imp method to complete datasets [19]. Other simple approaches tested, such as column-mean, column-median, gene-mean, and gene-median, handle missing values using the corresponding row/column mean or median [20].

### Performance evaluation of imputation algorithms

Comparison of different imputation methods is performed using the normalized root mean square error (NRMSE) index, which is calculated using the following formula:

(1) $$\text{NRMSE}=\sqrt{\frac{\mathrm{mean}{\left(y_{\mathrm{original}}-y_{\mathrm{imputed}}\right)}^{2}}{\mathrm{variance}(y_{\mathrm{original}})}}\text{,}$$

where $y_{\mathrm{original}}$ and $y_{\mathrm{imputed}}$ denote the original and imputed dataset, respectively. The NRMSE values range between zero and one, with smaller values indicative of better performance for evaluation [17].

### Efficiency of the imputation methods

To assess the efficiency of various approaches, all imputation methods were investigated for their ability to detect the crucial genes involved in cancers. Five well-known methods were applied to acquire the significant genes from the original and imputed datasets. These include the differential expression via distance summary (DEDS) [21], empirical Bayes analyses of microarrays (EBAM) [22], Limma [13], multiple testing (MULTTEST) [23], [24], and significance analysis of microarrays (SAM) [25], which are available as part of the Bioconductor project.

The chi-squared test for comparing the proportions of significant genes obtained is used to assess the strength of different imputation methods in recognizing important genes [26]. In our test, *p*_1_, *p*_2_, and *p*_3_ refer to the proportion of overlaps between significant genes detected from original data and those detected from the imputed data by LLS, MICE-CART, and column-mean, respectively. The null and alternative hypotheses are:

(2) $$\left\{\begin{array}{cc} H_{0}:\;p_{1}=p_{2}=p_{3} & \\ H_{1}:\;p_{i}\;\neq \;p_{j} & \text{for at least one pair}(i\text{,}j)\;\text{for}\;i\text{,}j\in \{1\text{,}2\text{,}3\}\text{.} \end{array}\right)$$

To test significant difference among *k* methods, a 2 × *k* contingency table is considered. In the table, the first row shows the overlaps between significant genes detected from original dataset and those detected from the imputed datasets, whereas the non-overlap between detected significant genes from original data and imputed datasets are determined in the second row. The chi-square test statistics is $\chi^{2}=\sum_{\mathrm{all}\;\mathrm{cells}}\frac{{\left(f_{o}-f_{e}\right)}^{2}}{f_{e}}$, where $f_{o}$ is the observed frequency in each cell of the contingency table, and $f_{e}$ is the expected frequency in the mentioned cell under the null hypothesis. The critical value is obtained from the quantile of $\chi^{2}$ distribution with (k-1)(2-1)=k-1 degrees of freedom at *α* level of significance, which is set as 0.05 in our test. If *P* < *α* (*P* denotes the *P* value), ***H*_0_**is rejected. This hypothesis test can be easily performed by the prop.test function in R [26], [27], [28].

### Pathway enrichment

Investigating differentially expressed genes is a common practice in detecting signatures or crucial genes involved in complex diseases such as cancer. However, we are more intrigued by discovering the prevalent roles of all genes rather than simply knowing what genes are involved in a complex disease [29]. Based on the assumption that genes do not act in isolation, and that complex diseases such as cancer are caused by perturbation of various pathways [30], [31], secondary data sources can be used to identify deregulated pathways during cancer progression. Gene pathway enrichment analysis is a powerful approach to address this problem by evaluating whether defined sets of genes are associated with particular biological processes.

Significant genes are detected by the SAM method, which derives the lists of differentially expressed genes with common, collective functions. Then, the set of significant genes from the original and two imputed datasets (MAR 10% and NMAR 30%), are enriched into pathways using ConsensusPathDB (*P* < 0.05). The enriched pathways are selected from KEGG, Wikipathways, Reactome, and SMPDB. Each pathway contains at least four significant genes. The role of these pathways in lung and rectal cancers is assessed through an extensive literature search.

## Results

**Figure 1:**
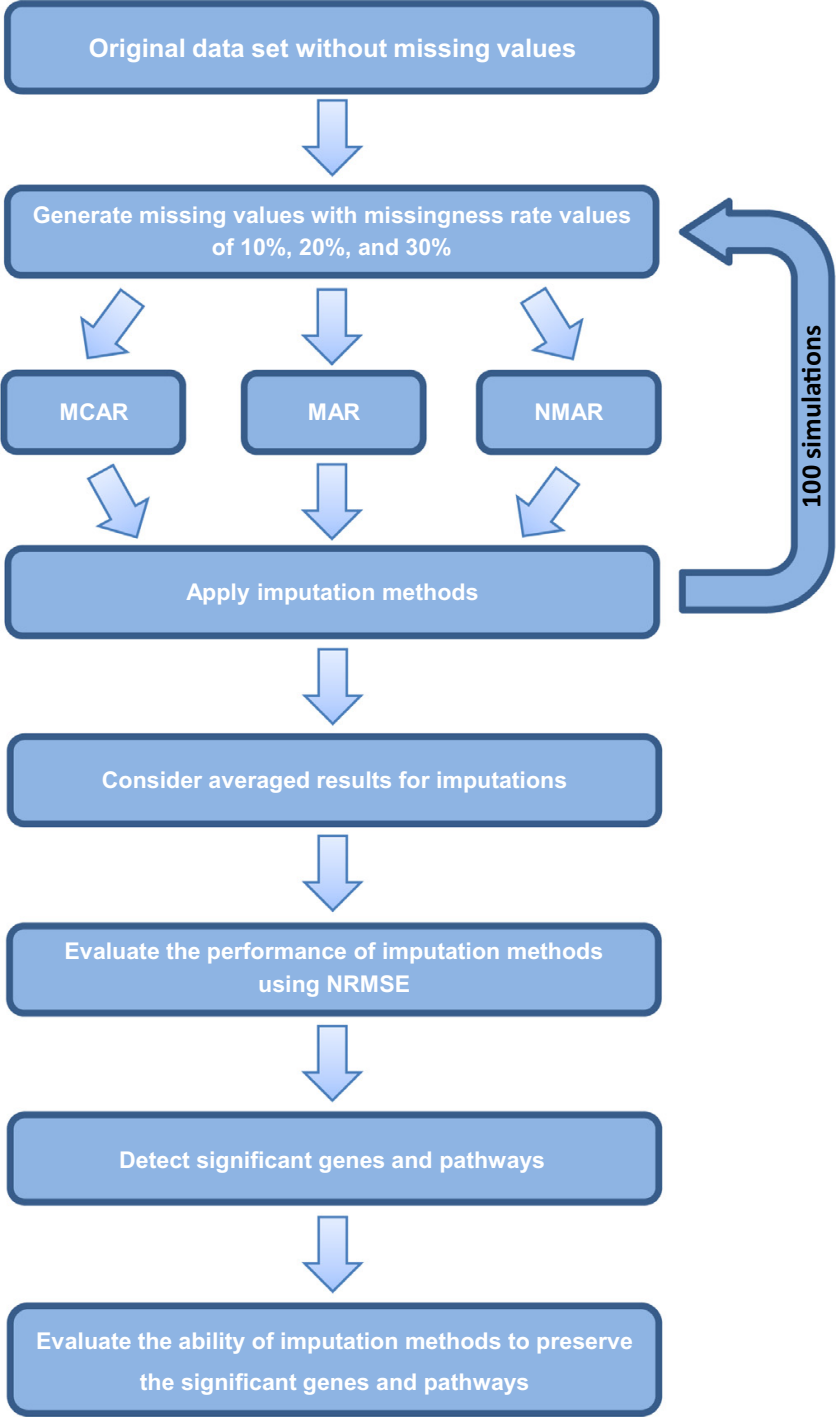
**Workflow for data analysis** 5% of the *N* genes are selected randomly from the original dataset to generate missing values. MCAR, MAR, and NMAR missingness mechanisms with the missingness rates 10%, 20%, and 30%, respectively, are considered. Then, ten imputation algorithms are applied to complete the datasets. For performance enhancement, the imputation procedures are repeated 100 times. The differences between the imputed and the original values are evaluated using the NRMSE index. Significant genes in the original and imputed datasets are detected using the SAM method and enriched into pathways. Finally, the ability of different imputation methods to preserve the significant genes and pathways is evaluated. MCAR, missing completely at random; MAR, missing at random (MAR); NMAR, not missing at random; NRMSE, normalized root mean square error.

In this study, to evaluate the sensitivity of the implemented imputation methods to the missingness mechanisms and rates, we randomly removed 10%, 20%, and 30% of genes via the MCAR, MAR or NMAR mechanisms. Then, we used different imputation approaches to impute the missing values. The imputation procedures were repeated a hundred times, and the mean and standard deviation of the NRMSE values were computed. Significant genes in the original and imputed datasets were detected using the SAM method and enriched into pathways. Finally, the ability of different imputation methods to preserve the significant genes and pathways was evaluated. A workflow of the analysis process is shown in Figure 1.

### Generating missing values for the *RFC2* gene in lung cancer dataset

**Figure 2:**
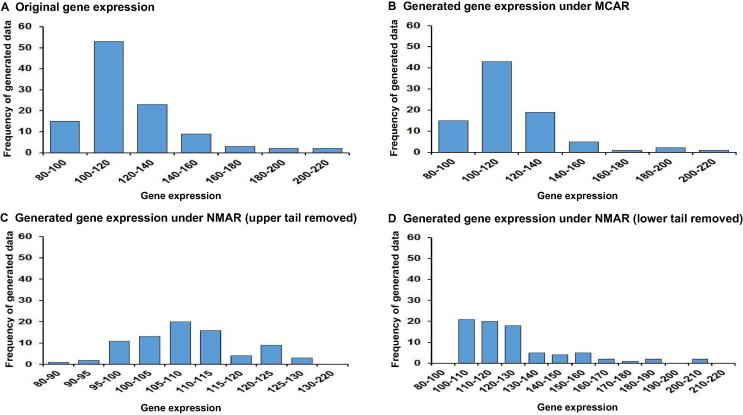
**Generating missing values in lung cancer dataset as exemplified for *RFC2* gene** **A.** The histogram of the gene expression for *RFC2* gene in the original lung cancer dataset. **B.** The histogram of the gene expression for *RFC2* gene in the generated lung cancer dataset after removing values under MCAR. Histograms of the values of gene expression after removing the upper and lower tails under NMAR, respectively, are shown in panels **C** and **D**, respectively. *RFC2*, replication factor C subunit 2; MCAR, missing completely at random; NMAR, not missing at random.

We used *RFC2* to exemplify the method for generating missing values. *RFC2* encodes the 40 kDa subunit of the replication factor C complex (also known as activator 1), which has been shown to be responsible for binding ATP and may help promote cell survival [32]. Also, previous studies have shown that *RFC2* is involved in three of the most significant pathways related to cell cycle regulation and DNA damage repair through 15 pan-cancer pathways relevant to drug response [33]. Missing values were generated for the lung cancer dataset using MCAR and NMAR mechanisms. As shown in Figure 2, after removing 20% of expression data via the MCAR mechanism, the expression profile for *RFC2* in lung cancer cells was similar to that of the original dataset (Figure 2A and B). In contrast, the histograms of gene expression data were altered after deleting 20% of the upper or lower tail of the values through the NMAR mechanism (Figure 2 C and D).

### Evaluating performance of imputation methods

**Figure 3:**
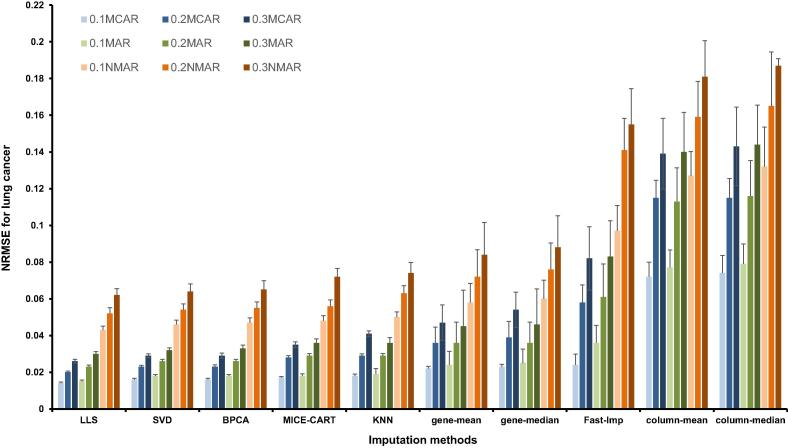
**NRMSE values of ten imputation algorithms for lung cancer** NRMSE values are plotted for the 10 imputation methods tested under different missingness mechanisms at the defined missingness rates. 0.1MCAR, 0.2MCAR, and 0.3MCAR denote the MCAR missingness mechanisms with 10%, 20%, and 30% missing percentages, respectively. Similar definition is also applied to the MAR and NMAR groups. NRMSE, normalized root mean square error; MCAR, missing completely at random; MAR, missing at random; NMAR, not missing at random. LLS, local least squares; SVD, singular value decomposition; BPCA, Bayesian principal component analysis; MICE-CART, multiple imputations by chained equations and classification and regression trees; KNN, K-nearest neighbor; Fast-Imp, fast imputation.

**Figure 4:**
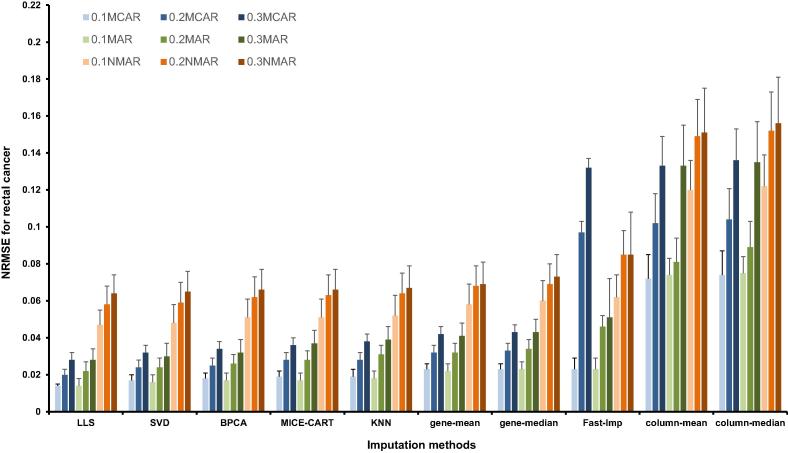
**NRMSE values of ten imputation algorithms for rectal cancer**

To evaluate the sensitivity of the implemented imputation methods to the missingness mechanisms and rates, we randomly removed 10%, 20%, and 30% of genes via the MCAR, MAR or NMAR mechanisms. Then, 10 different approaches were used to impute the missing values and the performance of the imputation methods tested was evaluated using NRMSE. As shown in Figure 3 for the lung cancer dataset, for all imputation methods, the mean NRMSE values tended to increase with higher missingness rates under the same missingness mechanism, whereas lower NRMSE values were obtained for MCAR and MAR mechanisms compared to NMAR. When comparing the NRMSE values obtained using different methods, LLS imputation approach performed the best with the lowest NRMSE values for each condition examined. Largely the similar trend was also observed for the rectal cancer dataset (Figure 4).

These imputation methods are classified into three groups based on the NRMSE values. The lowest NRMSE values were obtained when using LLS, SVD, and BPCA methods, whereas the highest NRMSE values were obtained for column-mean, column-median, and Fast-Imp methods. The remaining MICE-CART, gene-mean, gene-median, and KNN methods showed moderate NRMSE values. We thus chose one imputation method from each group for further analysis, which include LLS, MICE-CART, and column-mean.

### Detecting significant genes in imputed and original datasets

Two datasets were generated using the MAR missingness mechanism with 10% missingness rate and the NMAR missingness mechanism with 30% missingness rate, which were classified as group 1 and group 2, respectively. To compare the performance of different methods in detecting significant genes, we examined the overlaps between the significant genes from the aforementioned groups and those from the original dataset using methods SAM, DEDS, Limma, MULTTEST, and EBAM. It is expected that compared to group 2, the significant genes computed from group 1 would have more matches with the significant genes detected from the original dataset. Table 1 shows the overlaps between the detected significant genes according to the five aforementioned methods from the two generated groups of datasets and original datasets for lung and rectal cancers, respectively.

**Table 1:** **Overlaps between significant genes detected from the original datasets and those detected from the two generated dataset groups**

**Cancer type**	**Imputation method**	**Group**	**SAM**	**DEDS**	**Limma**	**MULTEST**	**EBAM**
Lung	LLS	1	484	483	453	473	481
		2	471	463	449	469	477

	MICE-CART	1	477	475	439	471	477
		2	468	476	446	469	465

	column-mean	1	473	469	438	463	473
		2	466	468	441	464	474

Rectal	LLS	1	996	993	985	993	988
		2	981	974	973	983	972

	MICE-CART	1	991	989	984	986	987
		2	978	972	966	987	962

	column-mean	1	989	986	981	973	974
		2	973	972	983	975	960

*Note*: Group 1 represents the datasets generated under the missing at random mechanism with 10% missingness rate and Group 2 represents the datasets generated under the not missing at random mechanism with 30% missingness rate. LLS, local least squares; MICE-CART, multiple imputations by chained equations and classification and regression trees; SAM, significance analysis of microarrays; DEDS, differential expression via distance summary; Limma, linear model for microarray analysis; MULTEST, multiple testing; EBAM, empirical Bayes analyses of microarrays.

Among five aforementioned methods, the SAM method satisfied this expectation the most and there were substantial similarities between the gene list of group 1 generated dataset and the original dataset for both cancer types imputed using different methods. Our findings are consistent with earlier findings about the detection of differential gene expression [34] and candidate loci [35] using the SAM method. Since SAM showed the best performance in detecting significant genes among the five methods for the two cancer types, SAM was selected for subsequent analysis in our study.

In total 490 and 1000 genes that putatively contribute to the lung and rectal cancer, respectively, were detected by applying SAM to the original datasets. We then applied SAM to the datasets generated with different missing mechanisms at the missingness rates of 0.1, 0.2, and 0.3, respectively, and imputed using different methods. The overlaps in significant genes detected between the imputed and original datasets for lung and rectal cancer were calculated. As shown in Table 2, the numbers of significant genes detected in the imputed and original datasets are nearly similar.

**Table 2:** **Common significant genes deciphered from the original datasets and those detected from the imputed datasets**

**Cancer type**	**Missingness mechanism**	**Missingness rate**	**Imputation method**			***P* value**
			**LLS**	**MICE-CART**	**column-mean**	
Lung	MCAR	0.1	485	479	477	0.161
		0.2	480	477	476	0.697
		0.3	478	476	475	0.839

	MAR	0.1	484	477	473	0.230
		0.2	479	476	469	0.170
		0.3	475	474	473	0.539

	NMAR	0.1	478	474	471	0.443
		0.2	475	472	469	0.595
		0.3	471	468	466	0.737

Rectal	MCAR	0.1	998	996	991	0.073
		0.2	996	995	990	0.194
		0.3	994	991	989	0.479

	MAR	0.1	996	991	989	0.194
		0.2	995	990	988	0.233
		0.3	993	986	984	0.160

	NMAR	0.1	988	987	983	0.602
		0.2	984	981	979	0.708
		0.3	981	978	973	0.478

*Note*: MCAR, missing completely at random; MAR, missing at random; NMAR, not missing at random; MICE-CART, multiple imputations by chained equations and classification and regression trees; LLS, local least squares.

We then tested the null hypothesis *H*_0_, no significant difference among different imputation methods, against the alternative hypothesis *H*_1_, a significant difference among different imputation methods, to detect the significant genes using the chi-squared test with equal proportions of mutual significant genes deciphered from original data and those detected from the imputed data by LLS, MICE-CART, and column-mean [26].

As shown in Table 2, *P* > 0.05 was found for all missingness mechanisms with different missingness rate, indicating that the three imputation methods examined, including LLS, MICE-CART, and column-mean, had a similar performance for the lung cancer dataset. Similarly, no significant differences in the performance of different imputation methods were detected for rectal cancer dataset either. Therefore, there is no significant difference among various imputation methods to preserve significant genes in lung and rectal cancer datasets.

### Identifying pathways enriched with significant genes

The progression of cancers can be attributed to the disturbance of various pathways [36]. To identify these pathways, the detected significant genes were enriched into pathways using ConsensusPathDB (*P* < 0.05) with each pathway containing at least 4 significant genes. For lung and rectal cancer datasets, 35 and 37 critical pathways were identified, respectively. The importance of pathways in lung and rectal cancers is illustrated through an extensive literature search (Table S1). Furthermore, significant genes from the aforementioned groups 1 and 2 were enriched in pathways as well. There is only one pathway different between these groups and the original dataset. For rectal cancer, only apoptosis modulation and signaling pathway [37] was detected to be significant in the groups 1 and 2. For lung cancer, the integrin-linked kinase signaling pathway [38] was selected as significant in the two groups.

## Discussions

Over the last few decades, a large amount of data have been collected via high-throughput technologies to decipher the differences between tumor and normal cells. These datasets have been successfully developed and used to identify target genes [30] causally involved in human cancer [39], [40]. Nevertheless, these datasets often suffer from missing values. Hence, imputation approaches have been developed to address this challenge [41]. Although various methods can be used to manage the missing values, outcomes could be quite different according to the datasets considered for each imputation method. Thus, selecting the appropriate imputation approach may affect the accuracy of the results obtained, as there is no imputation approach with perfect performance.

In the present study, we used a non-ignorable missingness mechanism (NMAR) and an ignorable mechanism (MCAR and MAR) to generate missing values within datasets and assessed the performance of each method for estimation of the missing values. The tested imputation methods are more effective at handling MCAR and MAR, than at handling NMAR missingness. Although many studies proposing how to deal with the non-ignorable mechanism have been published, the application of these methods in this interesting field of research could be improved. Our study shows that the LLS method is more appropriate for completing missing values in lung and rectal cancer datasets, based on the NRMSE values.

We also show that SAM can work effectively to detect important genes in lung and rectal cancers. All the five methods examined assume independent subject measurements (even within a single gene) to test the differential expression. Nevertheless, imputed data are not independent of the non-missing values, since the imputed value for a gene in a given subject is related to the non-missing values of the gene in other subjects. Currently, available tests may not be directly applicable for use, because the independence criteria need to be assumed. Building a statistical model to test the significance of a gene list accounting for the dependence between genes can be a challenging issue [42].

The resulting significant genes can be used to detect important pathways, with evidence available to support the role of candidate pathways in various cancer types [43], [44], [45], [46]. Furthermore, by selecting a 5% missingness rate in the original dataset, the results show that the imputation methods can detect significant genes and pathways similar to the original dataset. Finally, there is still uncertainty regarding the imputation methods to detect significant genes and pathways at different missingness rates, which needs to be addressed in further studies.

## Authors’ contributions

RA and ESA have conceived and designed the study. PK provided the data; RA and TB analyzed and interpreted the data. All authors were involved in manuscript writing, read and approved the final manuscript.

## Competing interests

The authors have declared that they had no competing interests.

## Supplementary material

Supplementary Table S1 The enriched pathways for the lung and rectal cancers
