# Supplementary material for: The Ability of Different Imputation Methods to Preserve the Significant Genes and Pathways in Cancer
Source: Genomics Proteomics Bioinformatics. 2017 Dec 13;15(6):396–404. doi: 10.1016/j.gpb.2017.08.003 (PMC5828654; doi:10.1016/j.gpb.2017.08.003)
Supplement: Supplementary Table S1 — The enriched pathways for the lung and rectal cancers [file mmc1.docx]

**Table S1 The enriched pathways for the lung and rectal cancers**

| **Cancer type** | **Pathway** | **Significant genes** | **PMID** |
| --- | --- | --- | --- |
| Lung | PLK1 signaling events | *TPX2*; *ECT2*; *BUB1B*; *CCNB1*; *CDC20*; *TUBG1*; *CDK1*; *CENPU*; *PRC1*; *KIF20A* | 23770244 |
|  | Platelet degranulation | *FIGF*; *PECAM1*; *HSPA5*; *TIMP1*; *F8*; *CFD*; *CD36*; *TTN*; *CLU*; *VWF*; *SELP*; *A2M*; *GAS6* | 22022533 |
|  | Response to elevated platelet cytosolic Ca^2+^ | *FIGF*; *PECAM1*; *TIMP1*; *HSPA5*; *F8*; *CFD*; *CD36*; *TTN*; *CLU*; *VWF*; *SELP*; *A2M*; *GAS6* | 1987767 |
|  | Hemostasis | *EHD2*; *TIMP1*; *DOCK6*; *CD36*; *KIF15*; *KIF11*; *ARRB1*; *A2M*; *KIF2C*; *CAV1*; *PECAM1*; *HSPA5*; *KIF4A*; *DOK2*; *TTN*; *ANGPT1*; *FIGF*; *PRKCH*; *JAM2*; *GAB2*; *DOCK4*; *GATA6*; *CLU*; *THBD*; *SERPINB6*; *F8*; *CFD*; *PDE2A*; *KLC1*; *VWF*; *GAS6*; *SELP*; *KIF20A* | 11372724 |
|  | APC/C: Cdc20 mediated degradation of mitotic | *CCNB1*; *MAD2L1*; *BUB1B*; *UBE2C*; *CDC20*; *CDK1*; *PTTG1* | 23151139 |
|  | Gastric cancer network 1 | *TPX2*; *ECT2*; *CENPF*; *UBE2C*; *NOTCH1*; *KIF15*; *TOP2A* | 25079317 |
|  | Retinoblastoma (RB) in cancer | *CCNB2*; *CCNB1*; *MCM6*; *RFC4*; *KIF4A*; *TTK*; *CDK1*; *RRM2*; *RPA3*; *SMARCA2*; *TOP2A* | 12204530 |
|  | Mitotic prometaphase | *CCNB2*; *CCNB1*; *MAD2L1*; *BUB1B*; *ZWINT*; *KNTC1*; *CDC20*; *CDK1*; *CENPF*; *CDCA8*; *CENPU*; *KIF2C* | 21918689 |
|  | Malaria - *Homo sapiens* (human) | *PECAM1*; *CD81*; *THBS2*; *CD36*; *HBB*; *HBA2*; *SELP* | 20657468 |
|  | Complement and coagulation cascades | *F8*; *C4BPA*; *CFD*; *CPB2*; *A2M*; *THBD*; *C7*; *VWF* | 15969748 |
|  | Human complement system | *VSIG4*; *SELP*; *CFD*; *CD93*; *SPP1*; *ICAM2*; *C4BPA*; *C7*; *A2M* | 25468776 |
|  | Platelet activation, signaling and aggregation | *FIGF*; *PECAM1*; *TIMP1*; *HSPA5*; *PRKCH*; *F8*; *CFD*; *GAB2*; *CD36*; *TTN*; *ARRB1*; *CLU*; *VWF*; *SELP*; *A2M*; *GAS6* | 24258815 |
|  | Factors involved in megakaryocyte development | *EHD2*; *DOCK6*; *DOCK4*; *KIF15*; *KIF11*; *KIF4A*; *GATA6*; *KLC1*; *KIF2C*; *KIF20A* | 11012231 |
|  | Extracellular matrix organization | *COLGALT2*; *PECAM1*; *COL11A1*; *TIMP1*; *EFEMP1*; *SPP1*; *MMP12*; *ADAMTS8*; *PCOLCE2*; *JAM2*; *PLOD2*; *MFAP4*; *COL1A2*; *ICAM2*; *MMP9*; *LAMC3*; *A2M*; *FBLN5* | 26805763 |
|  | Cell cycle checkpoints | *CCNB2*; *CCNB1*; *MAD2L1*; *MCM6*; *RFC4*; *UBE2C*; *BUB1B*; *RPA3*; *CDC20*; *CDK1*; *MCM2* | 15549093 |
|  | Beta1 integrin cell surface interactions | *MDK*; *COL11A1*; *JAM2*; *CD81*; *THBS2*; *COL1A2*; *SPP1* | 25707511 |
|  | Mitochondrial translation termination | *MRPL12*; *MRPL35*; *MRPL15*; *MRPS33*; *MRPL42*; *DAP3*; *MRPL13*; *MRPL3* | 12513701 |
|  | Glycolysis / gluconeogenesis - *Homo sapiens* | *GAPDH*; *ALDH2*; *PFKP*; *ENO1*; *TPI1*; *ADH1B*; *LDHA* | 21649891 |
|  | Purine nucleotides nucleosides metabolism | *NPR1*; *ATIC*; *NME1*; *NME1-NME2*; *HPRT1*; *PDE2A*; *PAPSS2*; *RRM2*; *PPAT* | 24667660 |
|  | M phase | *CCNB2*; *CCNB1*; *LMNB1*; *BUB1B*; *ZWINT*; *UBE2C*; *KNTC1*; *CDC20*; *CDK1*; *MAD2L1*; *CENPF*; *PTTG1*; *CENPU*; *CDCA8*; *KIF2C*; *KIF20A* | 25072848 |
|  | Mitochondrial translation | *MRPL12*; *MRPL13*; *MRPL15*; *MRPS33*; *MRPL42*; *DAP3*; *MRPL35*; *MRPL3* | 24675470 |
|  | Adherens junction - *Homo sapiens* (human) | *TGFBR2*; *WASF2*; *WASF3*; *LMO7*; *PTPRB*; *SORBS1*; *PTPRM* | 22674080 |
|  | Rho GTPases activate formins | *MAD2L1*; *BUB1B*; *ZWINT*; *KNTC1*; *CDC20*; *CENPF*; *CDCA8*; *CENPU*; *KIF2C* | 15588766 |
|  | Phase 1 - functionalization of compounds | *FMO2*; *CYP4B1*; *ALDH2*; *RXRA*; *MAOB*; *MAOA*; *ADH1B* | 23138267 |
|  | Metabolism of nucleotides | *ATIC*; *NME1*; *NME1-NME2*; *HPRT1*; *PPAT*; *DTYMK*; *RRM2* | 24486217 |
|  | Cell surface interactions at the vascular wall | *PECAM1*; *GAS6*; *JAM2*; *DOK2*; *THBD*; *ANGPT1*; *SELP*; *CAV1* | 7749329 |
|  | Cell adhesion molecules (CAMs) | *PECAM1*; *CDH3*; *JAM2*; *CDH5*; *CLDN18*; *CD34*; *ICAM2*; *PTPRM*; *SELP*; *CLDN5* | 15218541 |
|  | Phenytoin (antiarrhythmic) action pathway | *HSPA5*; *PDIA6*; *PDIA4*; *ATP1A2*; *CACNA2D2*; *AGTR1*; *TNNC1* | 1517643 |
|  | ECM-receptor interaction - *Homo sapiens* (human) | *COL11A1*; *COL1A2*; *THBS2*; *CD36*; *SPP1*; *LAMC3*; *VWF* | 22351925 |
|  | Validated targets of C-MYC transcriptional | *CCNB1*; *NME1*; *ENO1*; *NME1-NME2*; *GAPDH*; *HSPD1*; *MMP9*; *LDHA* | 9858526 |
|  | TGF beta signaling pathway | *TGFBR3*; *TGFBR2*; *CCNB2*; *MMP12*; *CDK1*; *NEDD4L*; *COL1A2*; *SPTBN1*; *CAV1* | 11586292 |
|  | Tight junction - *Homo sapiens* (human) | *MYH11*; *EPB41L2*; *JAM2*; *PRKCH*; *SPTAN1*; *CLDN18*; *RRAS*; *CLDN5*; *MYL9* | 11642738 |
|  | Vascular smooth muscle contraction | *MYH11*; *PRKCH*; *NPR1*; *RAMP3*; *RAMP2*; *PLA2G1B*; *AGTR1*; *MYL9* | 17907811 |
|  | Purine metabolism - *Homo sapiens* (human) | *NPR1*; *PAPSS2*; *NME1*; *NME1-NME2*; *HPRT1*; *PDE2A*; *ATIC*; *PPAT*; *RRM2*; *POLR2H* | 25476909 |
|  | Cell-cell communication | *ARHGEF6*; *SPTAN1*; *CLDN18*; *LIMS2*; *CDH3*; *CDH5*; *CLDN5*; *SPTBN1* | 25404879 |
| Rectal | Rho GTPases activate WASPs and WAVEs | *PTK2*; *NCK1*; *ARPC2*; *ABI1*; *MAPK1*; *ABL1*; *BAIAP2*; *NCKAP1*; *NCKIPSD* | 15588766 |
|  | Copper homeostasis | *BACE1*; *SOD1*; *PTEN*; *MT1H*; *SCO1*; *MT1E*; *MT1F*; *MT1G*; *MT1A*; *MT1B* | 18757361 |
|  | RNA transport - *Homo sapiens* (human) | *EIF3I*; *EIF3J*; *WIBG*; *ELAC1*; *NUP54*; *EIF4G3*; *EIF2B4*; *EIF2B5*; *PABPC4*; *NUP85*; *EIF3F*; *GEMIN7*; *NXT2*; *UPF3B*; *NXT1*; *EIF4EBP1*; *PAIP1*; *PNN*; *TGS1*; *NUP210* | 21796793 |
|  | Regulation of lipid metabolism by peroxisome proliferator-activated receptor alpha (PPARalpha) | *GRHL1*; *CPT2*; *PEX11A*; *ALAS1*; *TNFRSF21*; *FABP1*; *PLIN2*; *ACADM* | 22247890 |
|  | Fatty acid beta oxidation | *CPT1B*; *ACSL3*; *CPT2*; *SLC25A20*; *ACADVL*; *ACADM*; *GCDH* | 22621751 |
|  | Primary focal segmental glomerulosclerosis (FSGS) | *PODXL*; *PTK2*; *NCK1*; *ILK*; *CD2AP*; *CTNNB1*; *CD151*; *DAG1*; *NPHS1*; *PTPRO*; *CDH2* | 22187987 |
|  | Fatty acid degradation - *Homo sapiens* (human) | *CPT1B*; *ACSL3*; *CPT2*; *ACADVL*; *ADH1B*; *ACADSB*; *ACADM*; *GCDH* | 23791484 |
|  | JAK-STAT molecular variation 1 | *IL7R*; *IL13RA1*; *TNFSF10*; *CD40LG*; *GH1*; *IL10RB*; *IL5RA*; *CXCL8*; *IL2RB*; *IL6*; *IL9*; *IL1R2*; *IL10RA* | 23406773 |
|  | NOD-like receptor signaling pathway - *Homo sapiens* (human) | *CXCL1*; *IL6*; *CXCL2*; *CXCL8*; *BIRC3*; *MAPK14*; *MAPK1*; *HSP90AB1*; *NAIP* | 25635841 |
|  | Cell-cell communication | *CLDN10*; *CLDN3*; *NCK1*; *CLDN14*; *ILK*; *CD2AP*; *CTNNB1*; *CD151*; *PTK2*; *NPHS1*; *FLNA*; *CDH2*; *CLDN5*; *CLDN6*; *CLDN7* | 25404879 |
|  | TWEAK signaling pathway | *TNFSF12*; *TRAF3*; *BIRC3*; *MAPK14*; *IL6*; *CTNNB1*; *MAPK1* | 19201899 |
|  | Amoebiasis - *Homo sapiens* (human) | *CXCL1*; *RAB7B*; *PTK2*; *PLCB4*; *LAMA4*; *CXCL8*; *PRKCB*; *GNA14*; *IL6*; *C8B*; *COL3A1*; *IL1R2*; *SERPINB6* | 10524950 |
|  | Cytokine-cytokine receptor interaction - *Homo sapiens* (human) | *IL7R*; *TNFSF12*; *TNFSF10*; *CD40LG*; *IL10RA*; *IL10RB*; *ACVR2B*; *TNFRSF10B*; *IL1R2*; *AMHR2*; *CCL25*; *IL5RA*; *IL2RB*; *TNFRSF21*; *CXCL11*; *CXCL16*; *CXCL1*; *IL13RA1*; *CXCL2*; *GH1*; *CXCL6*; *CXCL8*; *KIT*; *IL6*; *IL9* | 19505916 |
|  | Cell junction organization | *CLDN10*; *CLDN3*; *CLDN14*; *ILK*; *CTNNB1*; *CD151*; *FLNA*; *CDH2*; *CLDN5*; *CLDN6*; *CLDN7* | 23458609 |
|  | Fatty acid, triacylglycerol, and ketone body metabolism | *ACADVL*; *HMGCL*; *ACSL3*; *CPT2*; *ELOVL4*; *TECR*; *SLC25A20*; *TGS1*; *FABP1*; *CPT1B*; *HSD17B3*; *ACADM* | 22009142 |
|  | Integrin-mediated cell adhesion | *GIT2*; *PTK2*; *ITGA11*; *CAPN1*; *ILK*; *CAPN7*; *MAPK1*; *MAPK4*; *SORBS1*; *PAK6*; *TNS1*; *CAPN9* | 14712234 |
|  | Drug metabolism - cytochrome P450 - *Homo sapiens* (human) | *FMO3*; *FMO4*; *UGT1A10*; *AOX1*; *ALDH3A1*; *MGST2*; *UGT2B15*; *ADH1B*; *ALDH1A3* | 16550168 |
|  | Acute myeloid leukemia - *Homo sapiens* (human) | *TCF7*; *SPI1*; *ZBTB16*; *CEBPA*; *KIT*; *MAPK1*; *EIF4EBP1*; *PML* | 18287387 |
|  | PPAR signaling pathway - *Homo sapiens* (human) | *CPT1B*; *ACSL3*; *CPT2*; *ILK*; *FABP5*; *FABP1*; *SORBS1*; *ACADM*; *SLC27A2* | 18584037 |
|  | Sphingolipid metabolism - *Homo sapiens* (human) | *DEGS1*; *UGCG*; *CERS4*; *ASAH2*; *CERK*; *SPTLC3*; *NEU2* | 16970943 |
|  | Angiogenesis overview | *NCK1*; *MAPK14*; *MAPK1*; *DAG1*; *GRB7*; *ANGPT1*; *MMP2*; *FGFR1* | 24659637 |
|  | Angiopoietin receptor Tie2-mediated signaling | *PTK2*; *NCK1*; *MAPK14*; *MAPK1*; *GRB7*; *ANGPT1*; *MMP2* | 22184396 |
|  | GPCR signaling-G alpha q | *TNFSF10*; *CD40LG*; *SMO*; *BMP15*; *BDNF*; *FZD5*; *FGF19*; *GAL*; *GRM8*; *EDN2*; *FGF16*; *CGB7*; *PLCB4*; *PRKCB*; *MLN*; *DLL4*; *NDP*; *INHA*; *GH1*; *CXCL8*; *IL6*; *VIP*; *MAPK1*; *IL9* | 24508914 |
|  | Salmonella infection - *Homo sapiens* (human) | *CXCL1*; *RAB7B*; *CXCL2*; *ARPC2*; *MAPK14*; *IL6*; *MAPK1*; *DYNC1LI2*; *CXCL8*; *FLNA* | 25196596 |
|  | Arrhythmogenic right ventricular cardiomyopathy (ARVC) - *Homo sapiens* (human) | *TCF7*; *ATP2A2*; *SGCG*; *DES*; *CTNNB1*; *ITGA11*; *DAG1*; *CACNG1*; *CDH2* | 9610536 |
|  | Fibroblast growth factor-1 | *RAB11A*; *PTK2*; *FIBP*; *CTNNB1*; *MAPK1*; *CSNK2A1*; *FLOT2*; *MAPK14*; *FGFR1* | 2162671 |
|  | Cell cycle - *Homo sapiens* (human) | *ORC5*; *CDC16*; *BUB1B*; *ORC6*; *CDC25A*; *CCND2*; *ORC3*; *ANAPC13*; *CDC14B*; *PLK1*; *ABL1*; *RBX1*; *ANAPC4* | 21990031 |
|  | Mineral absorption - *Homo sapiens* (human) | *ATP1B2*; *MT1H*; *MT1E*; *MT1F*; *MT1G*; *MT1A*; *MT1B* | 27634898 |
|  | Neural crest differentiation | *HDAC5*; *CTNNB1*; *FGF19*; *TBX6*; *RHOB*; *DLL4*; *HDAC10*; *MSX2*; *CDH2*; *GFAP*; *FGFR1* | 22261150 |
|  | Apoptosis-related network due to altered Notch3 in ovarian cancer | *IL7R*; *PTK2*; *F2R*; *TNFRSF10B*; *TNFRSF21*; *MAPK1*; *ABL1* | 24743243 |
|  | Rac1-Pak1-p38-MMP-2 pathway | *MAPK14*; *PTK2*; *NCK1*; *CTNNB1*; *MAPK1*; *EIF4EBP1*; *GRB7*; *ANGPT1* | 25595279 |
|  | Hemostasis | *PTK2*; *ATP2A2*; *ATP2A3*; *SCG3*; *CD84*; *MAPK14*; *RCOR1*; *ABL1*; *DOCK10*; *A2M*; *SERPINA5*; *F2R*; *SLC7A9*; *DGKQ*; *MFN1*; *GP1BB*; *MAPK1*; *ANGPT1*; *ITPK1*; *IL5RA*; *SOD1*; *LEFTY2*; *PRKCB*; *ATP1B2*; *SH2B2*; *SH2B3*; *MERTK*; *RHOB*; *SERPINB6*; *KIF3A*; *IL2RB*; *SLC16A3*; *SLC7A11*; *MFN2*; *GNA14*; *GNG5*; *GRB7*; *FLNA* | 11372724 |
|  | Urea cycle and metabolism of arginine, proline, glutamate, aspartate and asparagine | *ALDH3A1*; *NUP54*; *PARS2*; *P4HB*; *NUP62CL*; *GSTZ1*; *NUP85*; *MGST2*; *GATM*; *SLC25A20*; *NUP210* | 25476909 |
|  | Corticotropin-releasing hormone | *MAPK14*; *PTK2*; *KRT14*; *IVL*; *CXCL8*; *PRKCB*; *JUNB*; *CTNNB1*; *MAPK1*; *CRHR2* | 25015995 |
|  | Senescence and autophagy in cancer | *CXCL1*; *MAPK14*; *GABARAPL2*; *CXCL8*; *IL6*; *PTEN*; *ATG12*; *MAPK1*; *ATG7*; *MAP1LC3A*; *COL3A1* | 21878654 |
|  | p75(NTR)-mediated signaling | *CASP6*; *MAGEH1*; *BIRC3*; *SORT1*; *RHOB*; *BDNF*; *MMP3*; *FURIN* | 11359788 |
|  | Kit receptor | *IL7R*; *PRKCB*; *KIT*; *MAPK14*; *MAPK1*; *EIF4EBP1*; *GRB7*; *ABL1* | 17044945 |
|  | Degradation of the extracellular matrix | *BMP1*; *CTSB*; *MMP3*; *CMA1*; *CAPN1*; *MMP2*; *A2M*; *COL12A1*; *FURIN* | 9818170 |
